# Supplementary material for: Deep phenotypic profiling uncovers cryptic effects of antifilarial drugs
Source: PLoS Pathog. 2026 Jul 29;22(7):e1014485. doi: 10.1371/journal.ppat.1014485 (PMC13436727; doi:10.1371/journal.ppat.1014485)
Supplement: Supplementary file 1 [file ppat.1014485.s001.pdf]

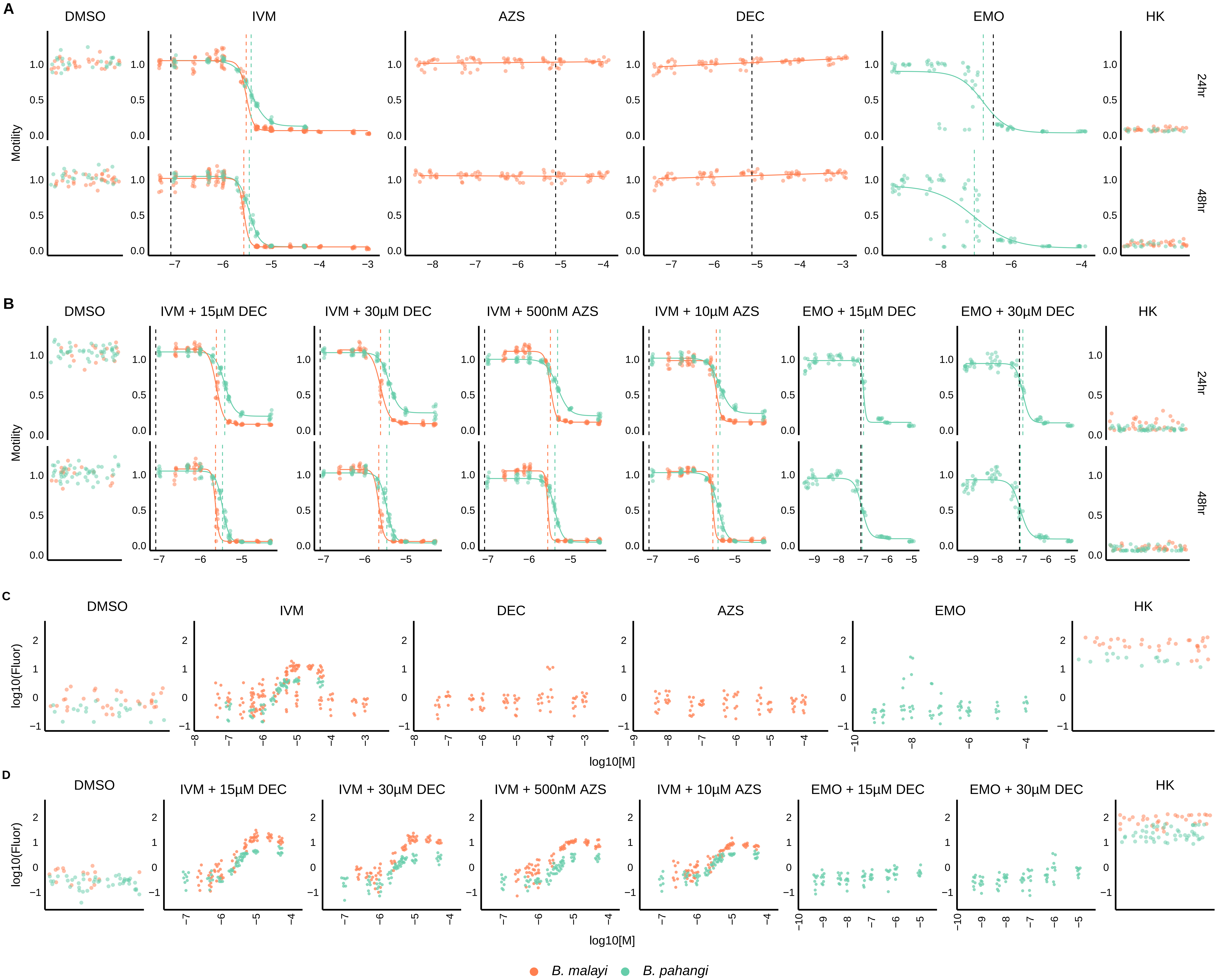
A
DMSO
IVM
AZS
DEC
EMO
HK
Motility
24hr
48hr
B
DMSO
IVM + 15µM DEC
IVM + 30µM DEC
IVM + 500nM AZS
IVM + 10µM AZS
EMO + 15µM DEC
EMO + 30µM DEC
HK
Motility
24hr
48hr
C
DMSO
IVM
DEC
AZS
EMO
HK
log10(Fluor)
log10[M]
D
DMSO
IVM + 15µM DEC
IVM + 30µM DEC
IVM + 500nM AZS
IVM + 10µM AZS
EMO + 15µM DEC
EMO + 30µM DEC
HK
log10(Fluor)
log10[M]
B. malayi
B. pahangi
