# Supplementary material for: Deep phenotypic profiling uncovers cryptic effects of antifilarial drugs
Source: PLoS Pathog. 2026 Jul 29;22(7):e1014485. doi: 10.1371/journal.ppat.1014485 (PMC13436727; doi:10.1371/journal.ppat.1014485)
Supplement: Supplementary file 2 [file ppat.1014485.s002.pdf]

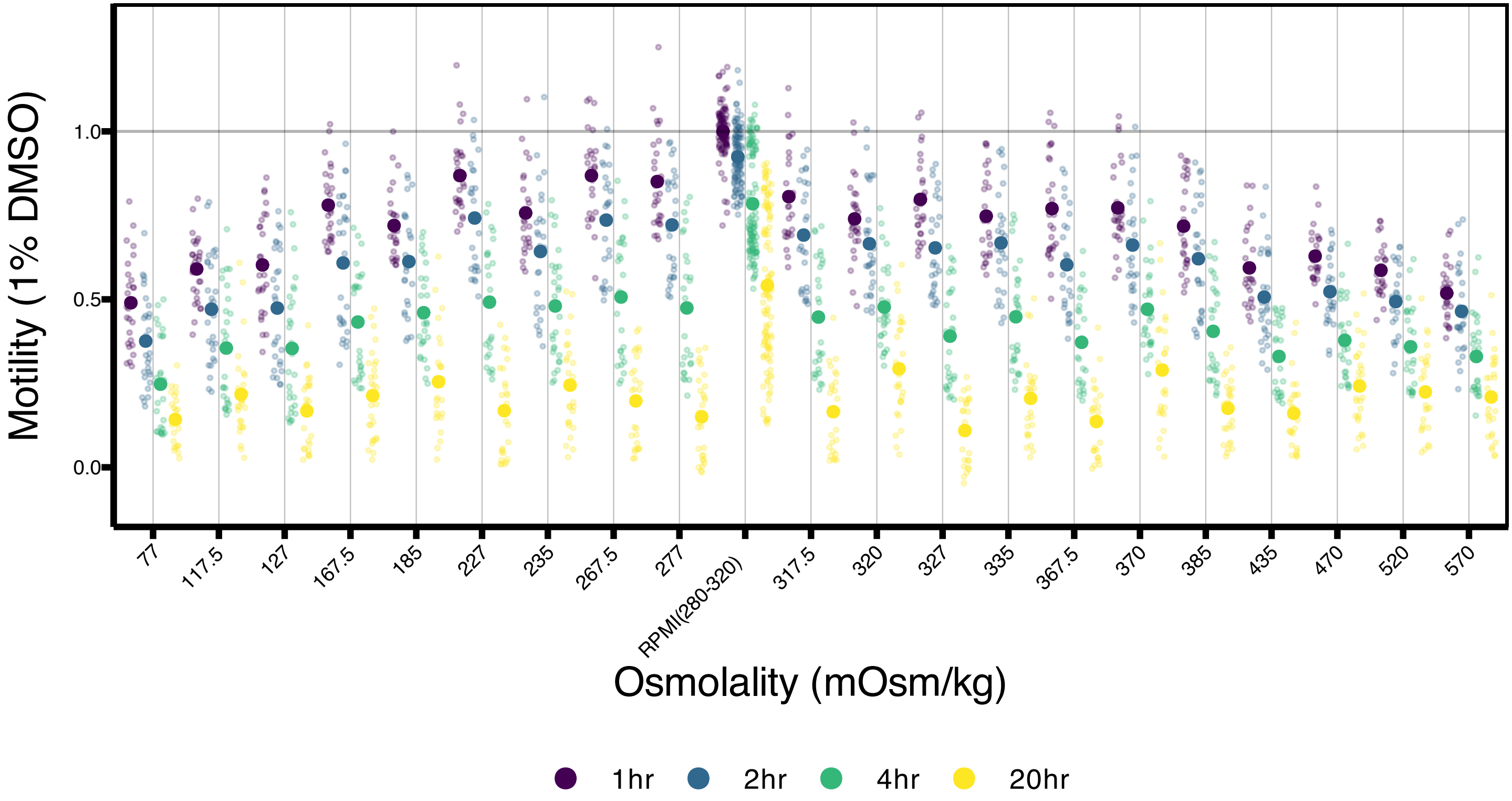

Motility (1% DMSO)
1.0
0.5
0.0
77
117.5
127
167.5
185
227
235
267.5
277
RPMI(280-320)
317.5
320
327
335
367.5
370
385
435
470
520
570
Osmolality (mOsm/kg)
1hr
2hr
4hr
20hr
